# Infrequent Detection of KI, WU and MC Polyomaviruses in Immunosuppressed Individuals with or without Progressive Multifocal Leukoencephalopathy

**DOI:** 10.1371/journal.pone.0016736

**Published:** 2011-03-16

**Authors:** Xin Dang, Seweryn Bialasiewicz, Michael D. Nissen, Theo P. Sloots, Igor J. Koralnik, Chen S. Tan

**Affiliations:** 1 Division of Viral Pathogenesis, Beth Israel Deaconess Medical Center, Boston, Massachusetts, United States of America; 2 Division of NeuroVirology, Department of Neurology, Beth Israel Deaconess Medical Center, Boston, Massachusetts, United States of America; 3 Queensland Pediatric Infectious Diseases Laboratory, Sir Albert Sakzewski Virus Research Centre, Brisbane, Australia; 4 Queensland Children's Medical Research Institute, Brisbane, Australia; 5 Pathology, Queensland Central, Brisbane, Australia; 6 Division of Infectious Diseases, Department of Medicine, Beth Israel Deaconess Medical Center, Boston, Massachusetts, United States of America; Johns Hopkins University - Bloomberg School of Public Health, United States of America

## Abstract

Conflicting prevalence of newly identified KI(KIPyV), WU(WUPyV) and Merkel Cell Carcinoma(MCPyV) polyomaviruses have been reported in progressive multifocal leukoencephalopathy(PML) patient samples, ranging from 0 to 14.3%. We analyzed the prevalence of these polyomaviruses in cerebrospinal fluid(CSF), peripheral blood mononuclear cells(PBMC), and bone marrow samples from PML patients, immunosuppressed individuals with or without HIV, and multiple sclerosis(MS) patients. Distinct PCR tests for KIPyV, WUPyV and MCPyV DNA performed in two independent laboratories detected low levels of MCPyV DNA only in 1/269 samples. The infrequent detections of these viruses in multiple samples from immunosuppressed individuals including those with PML suggest that their reactivation mechanisms may be different from that of JC polyomavirus (JCPyV) and that they do not play a role in the pathogenesis of PML.

## Introduction

Three new human polyomaviruses have been identified, KIPyV [1], WUPyV [2] and MCPyV [3]. Recent serological data have shown that in an adult population, seropositivity for KIPyV ranged from 63.3% to 70%, and for WUPyV ranged from 80%–100% [4]. While KIPyV and WUPyV were mainly detected in respiratory tract secretions in children, MCPyV was associated with a rare tumor of the skin called Merkel Cell Carcinoma. Further epidemiological investigations demonstrated that KI and WU PyV were predominantly found in patients with respiratory symptoms [5], [6], while MCPyV was also detected in the respiratory tract of children and immunosuppressed adults [5]. Therefore, a clear link between these polyomaviruses and a specific disease has yet to be established [7]. Several published reports investigated the possible pathogenic roles of the new polyomaviruses in blood, lymphoid tissue, and central nervous system (CNS) samples from immunosuppressed patients, including those with PML [7]–[14], showed discrepant results. Whereas one study reported detecting KIPyV and WUPyV in 14.3% of the brain samples from HIV-positive patients with PML [8], another study did not detect any of the three new polyomaviruses in the brain samples from HIV-positive patients with PML [9](Table 1). To clarify these conflicting results, we first performed a literature search and summarized all published findings in detection of KI, WU, MCPyVs in non respiratory samples of adult patients. Using different PCR methods at two distinct laboratories, we then screened for the three new polyomaviruses in 269 samples from 123 adult patients including HIV-positive with and without PML, HIV-negative with PML, multiple sclerosis (MS), other immunosuppressed patients and 2 HIV-negative immunocompetent patients with other neurological syndromes (ONS).

**Table 1:** Reported prevalence of new polyomaviruses in immunosurpressed and immunocompetent individuals.

Virus Name	HIV+/PML	HIV+ w/o PML	Other Immunosupressed	Hepatitis C+	CTRL	Specimen type	Reference
KIPyV	N/A	3/42 (7.1%)	N/A	N/A	1/55 (1.8%)	97 Lymphoid tissue	[10]
	N/A	0/6	N/A	N/A	0/54	60 CSF	[13]
	2/14 (14.3%)	6/25 (24%)	N/A	N/A	0/15	54 Brain tissue	[8]
	N/A	N/A	0/68	N/A	N/A	19 blood+49 lymph node	[15]
	0/7	N/A	N/A	N/A	N/A	7 Brain tissue	[9]
	N/A	1/100 (1%)	0/100	N/A	0/100	300 blood	[14]
	N/A	N/A	N/A	N/A	0/300	300 Brain tissue	[16]
	N/A	4/153 (2.6%)	N/A	N/A	N/A	N/A	[12]
WUPyV	N/A	3/42 (7.1%)	N/A	N/A	0/55	97 Lymphoid tissue	[10]
	N/A	1/6 (16.7%)	N/A	N/A	0/54	60 CSF	[13]
	2/14 (14.3%)	4/25 (16%)	N/A	N/A	0/15	54 Brain tissue	[8]
	N/A	N/A	0/68	N/A	N/A	19 blood+49 lymph node	[15]
	0/7	N/A	N/A	N/A	N/A	7 Brain tissue	[9]
	N/A	1/100 (1%)	0/100	N/A	0/100	300 blood	[14]
	N/A	10/121 (8.3%)	N/A	2/79 (2.5%)	0/120	320 Serum	[11]
	N/A	N/A	N/A	N/A	0/300	300 Brain tissue	[16]
	N/A	7/153 (4.5%)	N/A	N/A	N/A	N/A	[12]
MCPyV	N/A	0/42	N/A	N/A	1/55 (1.8%)	97 Lymphoid tissue	[10]
	N/A	0/6	N/A	N/A	0/54	60 CSF	[13]
	N/A	N/A	N/A	N/A	N/A	54 Brain tissue	[8]
	N/A	N/A	0/68	N/A	N/A	19 blood+49 lymph node	[15]
	0/7	N/A	N/A	N/A	N/A	7 Brain tissue	[9]
	N/A	N/A	N/A	N/A	N/A	300 blood	[14]
	N/A	N/A	N/A	N/A	0/300	300 Brain tissue	[16]

PML: progressive multifocal leukoencephalopathy; HIV+: HIV positive; w/o: without; CTRL: control; N/A: specimens not available; CSF: cerebral spinal fluid; KIPyV: KI polyomavirus; WUPyV: WU polyomavirus; MCPyV: Merckel cell carcinoma polyomavirus.

## Results

Laboratory 1 did not detect any of the 3 polyomaviruses DNA by PCR in all samples tested. Likewise, laboratory 2 also did not detect any positive sample for KIPyV and WUPyV among the 156 samples tested (Table S1, S2, S3, S4, S5, S6). There was one positive detection of MCPyV in the CSF from 36 samples in the immunosuppressed group (Table 2). This singular MCPyV positive produced a late cycle threshold value of 34.8, which is suggestive of a very low viral load.

**Table 2:** Summary of screening results of new polyomavirus from both laboratories.

Virus name	PML		HIV-positive	MS	Other Immunosuppressed	Hepatitis C Virus-positive	ONS	Result source
	HIV-positive	HIV-negative						
KIPyV	0/50	0/30	0/36	0/115	0/36	N/A	0/2	Laboratory 1
	0/50	0/30	0/36	0/2	0/36	N/A	0/2	Laboratory 2
WUPyV	0/50	0/30	0/36	0/115	0/36	N/A	0/2	Laboratory 1
	0/50	0/30	0/36	0/2	0/36	N/A	0/2	Laboratory 2
MCPyV	0/50	0/30	0/36	0/115	0/36	N/A	0/2	Laboratory 1
	0/50	0/30	0/36	0/2	1/36 (2.8%)	N/A	0/2	Laboratory 2

Total: 269 samples from 123 patients.

PML: progressive multifocal leukoencephalopathy; ONS: other neurological syndromes; N/A: specimens not available; MS: multiple sclerosis; KIPyV: KI polyomavirus; WUPyV: WU polyomavirus; MCPyV: Merckel cell carcinoma polyomavirus.

We have analyzed 8 published studies of detection of KI, WU and MC PyVs in CNS/blood/lymphoid tissue samples of adult patients (Table 1). 24 different primer sets were used in 21 different PCR conditions. Samples used in these studies included plasma, blood, CSF, brain biopsy and autopsy tissues, and autopsy lymphoid tissues. In all but one study, the healthy control group did not have any samples that contained detectable KI, WU, and MC PyV DNA. In the study by Sharp et al 1 healthy control had detectable KIPyV DNA and 1 healthy control had detectable MCPyV DNA [10]. However, unlike other reports, this study included HIV-positive, non AIDS patients in the healthy control group. In the “other (non-HIV) immunosuppressed” group, 3 studies reported no detection of any of the 3 new polyomaviruses, while one study detected WUPyV in 2.5% of patients with hepatitis C [11]. In the HIV-positive group, all studies detected at least one of the three polyomaviruses in at least one sample. Detection of KIPyV and WUPyV in HIV-positive PML patients were reported as 14.3% in one study and 0% in another. No study examined samples from the HIV-negative PML group, or patients with multiple sclerosis.

## Discussion

Our largely negative results suggest that the new human polyomaviruses WU, KI are not commonly found in the CNS, urine and blood of HIV-positive and MS patients, including those treated with natalizumab, and that immunosuppression does not result in replication and detection of these viruses in multiple compartments. Likewise, MCPyV was rarely detected in all populations. However, its detection in the CSF of an immunosuppressed patient may suggest neurotropic features of this virus, which is associated with a neuroendocrine tumor.

Overall, our data is in accordance with previously published studies, indicating that the novel human polyomaviruses are not typically detected in the CNS, blood and urine of HIV-positive and HIV-negative immunosuppresed patients (Table S2, S5). Therefore, whereas immunosuppression can cause reactivation of JCPyV and BKPyV resulting in increased detection of these viruses, immunosuppression does not seem to affect the reactivation and thus the detection of WU, KI, and MCPyV. A recent study did not find any difference in the prevalence of WU and KIPyV DNA detection in plasma of HIV-positive patients after grouping the patients by their CD4 counts [12]. By including HIV-negative immunosuppressed patients in our study (Table S2, S5), we have further explored whether immune dysfunction in addition to CD4 counts can affect the detection of these polyomaviruses.

Our results differed from the reported positive rate of KIPyV and WUPyV in immunosuppressed patients [8], [10], [13], [14]. Indeed, our data showed that the three new human polyomaviruses, aside from one exception, were not detected in CSF, brain biopsy, urine, bone marrow, and PBMC of PML patients with or without HIV and MS patients (Table S1, S2, S3). This differed from the study by Barzon et al [8] where 14.3% of the samples from PML patients had detectable KI and WU PyV, but is consistent with the findings of Focosi et al [9] and Maggi et al [15], who also reported no KIPyV and WUPyV DNA in PML and other immunosuppressed patients. Moreover, our results are also supported by the findings by Lam et al [16], who did not detect KIPyV, WUPyV and MCPyV in 300 brain samples from 30 individuals (Table S2). Lastly, our study is the first to include results from two different laboratories using different PCR methods, thereby confirming the study findings.

For the first time, we have also examined the prevalence of these new polyomaviruses in patients with multiple sclerosis (Table S3). The 113 samples from MS patients were collected both pre- and post-natalizumab treatment, providing an opportunity to investigate possible effects on DNA detection of new polyomavirus after blockade of alpha 4 integrin receptors. We have previously shown that up to 60 percent of these MS patients had evidence of JCPyV reactivation in blood samples during natalizumab treatment, while they all remained BK virus (BKPyV) negative [17]. In this current study we did not detect DNA from KIPyV, WUPyV, and MCPyV in the same samples in these patients. Although JCPyV reactivation in natalizumab treated MS patients remains controversial [18], results in this study suggested that there was no co-reactivation of new PyVs with JCPyV in our cohort of MS patients treated with natalizumab. These results also provide evidences that the pathogenesis of these three new polyomaviruses may be more closely related to BKPyV than JCPyV.

Our screening for DNA of MCPyV was positive in only 1/36 (2.8%) CSF samples from the HIV-negative immunosuppressed group without PML (Table 2), which differed from the results from another study [15] . Absence of KIPyV and WUPyV also differed from data published by other groups [8], [10], [13], [14]. These conflicting results may be due to geographical diversity in the patient or in the viral populations, and the different laboratory methods used in the various studies. Indeed, the sensitivity of PCR assays can be affected in part by sequence variation and methods. Collaborative investigations and standardization of detection methods will be important to help understand the true prevalence of new polyomaviruses in clinical samples from different geographic origins.

Lastly, our results and other studies did not find any clear associations between KI, WU, and MC PyV detection in human samples and immune suppression due to HIV, or immune dysregulation due to MS and its treatment. In our study, WU, KI, and MC PyV were generally not detected in a wide range of immunosuppressed patient samples, particularly within the CNS and blood. These results suggest that in addition to different viral tropism, the mechanisms of viral pathogenesis in latency and reactivation differs significantly among the various human polyomaviruses.

## Materials and Methods

Samples were collected from patients seen at Beth Israel Deaconess Medical Center, Boston, MA. Study was approved by the Institutional Review Board.

A total of 269 samples were collected from 123 patients from 6 groups: HIV-positive with PML, HIV-negative with PML, HIV-positive, MS, other immunosuppressed patients and patients with ONS (Tables S1, S2, S3, S4, S5, S6). Samples contained 8 brain autopsy tissues from 6 patients (6 from 5 HIV-positive patients with PML, 2 from a patient with chronic lymphocytic leukemia(CLL)), 62 CSF samples from 59 patients (30 from 29 HIV-positive patients with PML, 12 from 12 HIV-negative patients with PML, 6 from 6 HIV-positive patients, 10 from 10 patients immunosuppressed by other diseases and 2 from 2 MS patients), 13 whole blood samples from 13 patients (2 from 2 HIV-negative patients with PML, 2 from 2 HIV-positive lymphoma patients, 3 from lymphoma patients, 2 from 2 patients with monoclonal gammopathy of undetermined significance(MGUS), 1 from a patient with thrombocytosis, 1 from a HIV-positive patient with anemia, 1 from a patient with Leshmaniasis, 1 from a CLL patient), 68 PBMC samples from 49 patients (14 from 14 HIV-positive patients with PML, 10 from 10 HIV-negative patients with PML, 7 from 7 HIV-positive patients, 19 from MS patients prior to natalizumab treatment, 18 from MS patients after natalizumab treatment). 47 blood plasma samples from 28 patients (3 from 3 HIV-negative patients with PML, 3 from 3 HIV-negative lymphoma patients, 1 from 1 HIV-negative MGUS patient, 1 from 1 HIV-positive lymphoma patient, 1 from 1 HIV-positive patient, 19 from MS patients prior to natalizumab treatment, 19 from MS patients after natalizumab treatment). 13 bone marrow samples from 13 patients (2 from 2 HIV-negative patients with PML, 2 from 2 MGUS patients, 2 from HIV-negative lymphoma patients, 1 from HIV-negative Leshmaniasis patient, 1 from HIV-negative CLL patient, 3 from HIV-positive lymphoma patients, 1 from 1 HIV-positive patient with thrombocytosis, 1 from 1 HIV-positive patient with anemia), 8 bone marrow plasma samples from 8 patients (2 from 2 HIV-negative patients with PML, 3 from 3 HIV-negative lymphoma patients, 1 from 1 HIV-negative MGUS patient, 1 from 1 HIV-positive patient with thrombocytosis, 1 from 1 HIV-positive lymphoma patient), 1 bone marrow PBMC sample from 1 HIV-negative lymphoma patient, and 47 urine samples from 28 patients (3 from 3 HIV-negative lymphoma patients, 2 from 2 HIV-negative MGUS patients, 1 from 1 HIV-negative patient with Leshmaniasis, 1 from 1 HIV-positive patient with anemia, 1 from 1 HIV-positive patient with thrombocytosis, 1 from HIV-positive lymphoma patient, 19 from 19 MS patients prior to natalizumab treatment, and 19 from 19 MS patients after natalizumab treatment). Two CSF samples collected from 2 HIV-negative immunocompetent patients with ONS were also included. All samples were stored at either −20°C or −80°C. All 269 samples were tested for the new polyomaviruses in laboratory 1. Of the 269 samples, 153 samples were transferred to laboratory 2 for testing.

### DNA extraction

QIAamp DNA Blood Mini Kit (QIAGEN, Valencia, CA) was used to extract DNA. DNA concentration was determined by NanoDrop ND-1000 spectrophotometer (Thermo Scientific, Waltham, MA).

### PCR reaction

Laboratory 1 used primers POLVP1-39F(5′-AAGGCCAAGAAGTCAAGTTC-3′, KIPyV(1536-1555) and POLVP1-363R(5′-ACACTCACTAACTTGATTTGG-3′, KIPyV(1860-1840) reverse compliment(RC)) [1] for KIPyV AG0048(5′-TGTTTTTCAAGTATGTTGCATCC-3′, WUPyV 2822-2844) and AG0049(5′-CACCCAAAAGACACTTAAAAGAAA-3′, WUPyV 3065-3042 RC) [2] for WUPyV and primers LT3F571 (5′-TTGTCTCGCCAGCATTGTAG-3′), MCPyV (571-590) and LT3R879 (5′-ATATAGGGGCCTCGTCAACC-3′. MCPyV 879-860 RC) [3] for MCPyV were used in a qualitative PCR assays by laboratory 1 as previously described [1], [2], [3]. In each reaction, 0.5 ug of DNA was used. For sample where DNA quantification is not possible, 10 ul of the extraction mixture was used per reaction

A triplex real time PCR was used by laboratory 2 to detect KIPyV and WUPyV. A total of three sets of primer pairs and probes were used in the reaction: set # 1: Primer KI-D-F(5′-CACAGGTGGTTTTCTATAAATTTTGTACTT-3′. KIPyV 4524-4553), primer KI-D-R(5′-GAAGCAGTGGGATGTATGCATTC-3′, KIPyV 4635-4613 RC), probe KI-D-Pr (5′-HEX-TGCATTGGCATTCGTGATTGTAGCCA-BHQ1-3′, KIPyV 4586-4611); set # 2: primer KI-E-F (5′-GAACTTCTACTGTCCTTGACACAGGTA-3′, KIPyV 247-273), primer KI-E-R (5′-GGATTAGAACTTACAGTCTTAGCATTTCAG-3′, KIPyV 345-316 RC), probe KI-E-Pr (5′-Quasar 670-TGGGAAACATCCGGTTTCCTCTCACTTCC-BHQ2-3′, KIPyV 316-345); set # 3: primer WU-F-F (5′-GCCGACAGCCGTTGGATATA-3′, WUPyV 531-550), primer WU-F-R (5′-TTTCAGGCACAGCAAGCAAT-3′, WUPyV 601-582 RC), probe WU-F-Pr (5′-FAM-AGGGTCACCATTTTTATTTCAGATGGGCA-BHQ1-3′, WUPyV 552-580). For MCPyV detection, primer MCV-1.1-F(5′-AGCTCAGAAGTGACTTCTCTATGTTTGA-3′, MCPyV 398-425), primer MCV-1.1-R(5′-ACAATGCTGGCGAGACAACT-3′, MCPyV 588-569 RC) and probe MCV-1.1-Prb(5′-JOE-TTTGCAGAGGTCCTGGGTGCATG-BHQ1-3′, MCPyV 503-525) were used in the first round detection and then confirmed by MCV2.0 primer and probe set (primer MCPyV-2.0-4367F: 5′-GGCAGCATCCCGGCTTA-3′, primer MCPyV-2.0-4399R: 5′-CCAAAAAGAAAAGCATCATCCA-3′, probe MCPyV-2.0-4371-Prb: 5′-FAM-ATACATTGCCTTTTGGGTGTTTT-BHQ1-3′) as described previously [6]. The PCR reactions consisted of 12.5 µl Quantitect Probe PCR Mix (QIAGEN, Australia), 10 pmol of each primer, 4 pmol of each probe and 2 µl of template in a 25 µl final reaction, which was performed in either a Rotorgene 3000 or Rotorgene 6000(Corbett Research, Sydney, Australia) under the following conditions: 15 minutes incubation at 95°C, followed by 50 cycles of 95°C for 15 seconds and 60°C for 1 minute. PCR sensitivity was 10 copies per reaction.

Plasmids containing KIPyV, WUPyV and MCPyV genome (generous gifts from Dr. Tobias Allander of Karolinska Institute, Dr. David Wang of Washington University School of Medicine and Dr. Huichen Feng of University of Pittsburgh) were used as positive controls in Laboratory 1, while Laboratory 2 used clinical samples containing WU, KI and MC PyV DNA confirmed by sequencing.

### Literature search

PubMed searches were performed using terms “human polyomavirus” and “detection”. Only papers in English language and studies with adult patients were reviewed, and studies using respiratory samples were excluded.

## Supporting Information

Table S1 **Summary of all samples.** All 269 samples were categorized into PML, MS and non-PML/non-MS groups in Table S1A. All 269 samples were categorized into immunosuppressed and immunocompetent groups in Table S1B.
(DOC)

Table S2 **Samples from PML patients (80 samples from 61 patients).**
(DOC)

Table S3 **Samples from MS patients (115 samples from 21 patients).**
(DOC)

Table S4 **Samples from non-PML, non-MS patients (74 samples from 41 patients).**
(DOC)

Table S5 **Samples from Immunosuppressed patients (113 samples from 90 patients).**
(DOC)

Table S6 **Samples from Immunocompetent patients (156 samples from 33 patients).**
(DOC)

## References

[1] Allander T, Andreasson K, Gupta S, Bjerkner A, Bogdanovic G (2007). Identification of a third human polyomavirus.. J Virol.

[2] Gaynor AM, Nissen MD, Whiley DM, Mackay IM, Lambert SB (2007). Identification of a novel polyomavirus from patients with acute respiratory tract infections.. PLoS Pathog.

[3] Feng H, Shuda M, Chang Y, Moore PS (2008). Clonal integration of a polyomavirus in human Merkel cell carcinoma.. Science.

[4] Nguyen NL, Le BM, Wang D (2009). Serologic evidence of frequent human infection with WU and KI polyomaviruses.. Emerg Infect Dis.

[5] Bialasiewicz S, Whiley DM, Lambert SB, Nissen MD, Sloots TP (2009). Detection of BK, JC, WU, or KI polyomaviruses in faecal, urine, blood, cerebrospinal fluid and respiratory samples.. J Clin Virol.

[6] Bialasiewicz S, Lambert SB, Whiley DM, Nissen MD, Sloots TP (2009). Merkel cell polyomavirus DNA in respiratory specimens from children and adults.. Emerg Infect Dis.

[7] Norja P, Ubillos I, Templeton K, Simmonds P (2007). No evidence for an association between infections with WU and KI polyomaviruses and respiratory disease.. J Clin Virol.

[8] Barzon L, Squarzon L, Militello V, Trevisan M, Porzionato A (2009). WU and KI polyomaviruses in the brains of HIV-positive patients with and without progressive multifocal leukoencephalopathy.. J Infect Dis.

[9] Focosi D, Maggi F, Andreoli E, Lanini L, Ceccherini-Nelli L (2009). Polyomaviruses other than JCV are not detected in progressive multifocal leukoencephalopathy.. J Clin Virol.

[10] Sharp CP, Norja P, Anthony I, Bell JE, Simmonds P (2009). Reactivation and mutation of newly discovered WU, KI, and Merkel cell carcinoma polyomaviruses in immunosuppressed individuals.. J Infect Dis.

[11] Miller MA, Weibel C, Ferguson D, Landry ML, Kahn JS (2009). WU polyomavirus in patients infected with HIV or hepatitis C virus, Connecticut, USA, 2007.. Emerg Infect Dis.

[12] Babakir-Mina M, Ciccozzi M, Farchi F, Bergallo M, Cavallo R (2010). KI and WU polyomaviruses and CD4+ cell counts in HIV-1-infected patients, Italy.. Emerg Infect Dis.

[13] Barzon L, Squarzon L, Pacenti M, Scotton PG, Palu G (2009). Detection of WU polyomavirus in cerebrospinal fluid specimen from a patient with AIDS and suspected progressive multifocal leukoencephalopathy.. J Infect Dis.

[14] Barzon L, Squarzon L, Militello V, Trevisan M, Palu G (2009). Human KI and WU polyomavirus infection in immunocompromised subjects.. J Clin Virol.

[15] Maggi F, Focosi D, Ciancia E, Andreoli E, Lanini L (2010). WU and KI polyomaviruses remain orphans in adults.. J Infect Dis.

[16] Lam WY, Leung BW, Chu IM, Chan AC, Ng HK (2010). Survey for the presence of BK, JC, KI, WU and Merkel cell polyomaviruses in human brain tissues.. J Clin Virol.

[17] Chen Y, Bord E, Tompkins T, Miller J, Tan CS (2009). Asymptomatic reactivation of JC virus in patients treated with natalizumab.. N Engl J Med.

[18] Gorelik L, Goelz S, Sandrock AW, De Gascun CF, Lonergan RM (2009). Asymptomatic Reactivation of JC Virus in Patients Treated with Natalizumab.. N Engl J Med.

