# Supplementary material for: Infrequent Detection of KI, WU and MC Polyomaviruses in Immunosuppressed Individuals with or without Progressive Multifocal Leukoencephalopathy
Source: PLoS One. 2011 Mar 16;6(3):e16736. doi: 10.1371/journal.pone.0016736 (PMC3059210; doi:10.1371/journal.pone.0016736)
Supplement: Table S1 — Summary of all samples. All 269 samples were categorized into PML, MS and non-PML/non-MS groups in Table S1A. All 269 samples were categorized into immunosuppressed and immunocompetent groups in Table S1B. (DOC) [file pone.0016736.s001.doc]

**Table S1**: Summary of all samples. All 269 samples were categorized into PML, MS and non-PML/non-MS groups in Table S1A. All 269 samples were categorized into immunosuppressed and immunocompetent groups in Table S1B.

Table S1A

| Virus name | PML | MS | non-PML, non-MS | Result source |
| --- | --- | --- | --- | --- |
| KIPyV | 0/80 | 0/115 | 0/74 | Lab 1 |
|  | 0/80 | 0/2 | 0/74 | Lab 2 |
| WUPyV | 0/80 | 0/115 | 0/74 | Lab 1 |
|  | 0/80 | 0/2 | 0/74 | Lab 2 |
| MCPyV | 0/80 | 0/115 | 0/74 | Lab 1 |
|  | 0/80 | 0/2 | **1/74** | Lab 2 |

Table S1B

| Virus name | Immunosuppressed | Immunocompetent | Result source |
| --- | --- | --- | --- |
| KIPyV | 0/113 | 0/156 | Lab 1 |
|  | 0/113 | 0/43 | Lab 2 |
| WUPyV | 0/113 | 0/156 | Lab 1 |
|  | 0/113 | 0/43 | Lab 2 |
| MCPyV | 0/113 | 0/156 | Lab 1 |
|  | **1/113** | 0/43 | Lab 2 |

PML: progressive multifocal leukoencephalopathy; MS: multiple sclerosis; KIPyV: KI polyomavirus; WUPyV: WU polyomavirus; MCPyV: Merckel cell carcinoma polyomavirus.
