# Supplementary material for: Infrequent Detection of KI, WU and MC Polyomaviruses in Immunosuppressed Individuals with or without Progressive Multifocal Leukoencephalopathy
Source: PLoS One. 2011 Mar 16;6(3):e16736. doi: 10.1371/journal.pone.0016736 (PMC3059210; doi:10.1371/journal.pone.0016736)
Supplement: Table S2 — Samples from PML patients (80 samples from 61 patients). (DOC) [file pone.0016736.s002.doc]

| **Table S2**: Samples from PML patients (80 samples from 61 patients) | | | | | | | | | | | |
| --- | --- | --- | --- | --- | --- | --- | --- | --- | --- | --- | --- |
| Virus name | HIV+/PML | | | HIV-/PML | | | | | | | Result source |
|  | Brain | CSF | PBMC | CSF | PBMC | Bone marrow | Whole blood | BM plasma | Blood plasma | Cell free blood plasma |  |
| KIPyV | 0/6 | 0/30 | 0/14 | 0/12 | 0/9 | 0/2 | 0/2 | 0/2 | 0/2 | 0/1 | Lab 1 |
|  | 0/6 | 0/30 | 0/14 | 0/12 | 0/9 | 0/2 | 0/2 | 0/2 | 0/2 | 0/1 | Lab 2 |
| WUPyV | 0/6 | 0/30 | 0/14 | 0/12 | 0/9 | 0/2 | 0/2 | 0/2 | 0/2 | 0/1 | Lab 1 |
|  | 0/6 | 0/30 | 0/14 | 0/12 | 0/9 | 0/2 | 0/2 | 0/2 | 0/2 | 0/1 | Lab 2 |
| MCPyV | 0/6 | 0/30 | 0/14 | 0/12 | 0/9 | 0/2 | 0/2 | 0/2 | 0/2 | 0/1 | Lab 1 |
|  | 0/6 | 0/30 | 0/14 | 0/12 | 0/9 | 0/2 | 0/2 | 0/2 | 0/2 | 0/1 | Lab 2 |

HIV+: HIV positive; HIV-: HIV negative; PML: progressive multifocal leukoencephalopathy; CSF: cerebral spinal fluid; PBMC: peripheral blood mononuclear cells; N/A: not available; BM: bone marrow; KIPyV: KI polyomavirus; WUPyV: WU polyomavirus; MCPyV: Merckel cell carcinoma polyomavirus.
