# Supplementary material for: Infrequent Detection of KI, WU and MC Polyomaviruses in Immunosuppressed Individuals with or without Progressive Multifocal Leukoencephalopathy
Source: PLoS One. 2011 Mar 16;6(3):e16736. doi: 10.1371/journal.pone.0016736 (PMC3059210; doi:10.1371/journal.pone.0016736)
Supplement: Table S3 — Samples from MS patients (115 samples from 21 patients). (DOC) [file pone.0016736.s003.doc]

| **Table S3**: Samples from MS patients (115 samples from 21 patients) | | | | | | | | |
| --- | --- | --- | --- | --- | --- | --- | --- | --- |
| Virus Name | MS w/o natalizumab | | | | MS with natalizumab | | | Result source |
|  | CSF | PBMC | Plasma | Urine | PBMC | Plasma | Urine |  |
| KIPyV | 0/2 | 0/19 | 0/19 | 0/19 | 0/18 | 0/19 | 0/19 | Lab 1 |
|  | 0/2 | N/A | N/A | N/A | N/A | N/A | N/A | Lab 2 |
| WUPyV | 0/2 | 0/19 | 0/19 | 0/19 | 0/18 | 0/19 | 0/19 | Lab 1 |
|  | 0/2 | N/A | N/A | N/A | N/A | N/A | N/A | Lab 2 |
| MCPyV | 0/2 | 0/19 | 0/19 | 0/19 | 0/18 | 0/19 | 0/19 | Lab 1 |
|  | 0/2 | N/A | N/A | N/A | N/A | N/A | N/A | Lab 2 |

CSF: cerebral spinal fluid; MS: multiple sclerosis; w/o: without; PBMC: peripheral blood mononuclear cells; N/A: not available; BM: bone marrow; KIPyV: KI polyomavirus; WUPyV: WU polyomavirus; MCPyV: Merckel cell carcinoma polyomavirus.
