# Supplementary material for: Infrequent Detection of KI, WU and MC Polyomaviruses in Immunosuppressed Individuals with or without Progressive Multifocal Leukoencephalopathy
Source: PLoS One. 2011 Mar 16;6(3):e16736. doi: 10.1371/journal.pone.0016736 (PMC3059210; doi:10.1371/journal.pone.0016736)
Supplement: Table S4 — Samples from non-PML, non-MS patients (74 samples from 41 patients). (DOC) [file pone.0016736.s004.doc]

| **Table S4**: Samples from non-PML, non-MS patients (74 samples from 41 patients) | | | | | | | | | | | |
| --- | --- | --- | --- | --- | --- | --- | --- | --- | --- | --- | --- |
| Virus Name | Brain | CSF | PBMC | Plasma | Bone marrow | Whole blood | BM plasma | Blood plasma | BM PBMC | Urine | Result source |
| KIPyV | 0/2 | 0/20 | 0/8 | 0/1 | 0/11 | 0/11 | 0/6 | 0/5 | 0/1 | 0/9 | Lab 1 |
|  | 0/2 | 0/20 | 0/8 | 0/1 | 0/11 | 0/11 | 0/6 | 0/5 | 0/1 | 0/9 | Lab 2 |
| WUPyV | 0/2 | 0/20 | 0/8 | 0/1 | 0/11 | 0/11 | 0/6 | 0/5 | 0/1 | 0/9 | Lab 1 |
|  | 0/2 | 0/20 | 0/8 | 0/1 | 0/11 | 0/11 | 0/6 | 0/5 | 0/1 | 0/9 | Lab 2 |
| MCPyV | 0/2 | 0/20 | 0/8 | 0/1 | 0/11 | 0/11 | 0/6 | 0/5 | 0/1 | 0/9 | Lab 1 |
|  | 0/2 | **1/20** | 0/8 | 0/1 | 0/11 | 0/11 | 0/6 | 0/5 | 0/1 | 0/9 | Lab 2 |

CSF: cerebral spinal fluid; PBMC: peripheral blood mononuclear cells; N/A: not available; BM: bone marrow; KIPyV: KI polyomavirus; WUPyV: WU polyomavirus; MCPyV: Merckel cell carcinoma polyomavirus.
