# Supplementary material for: Infrequent Detection of KI, WU and MC Polyomaviruses in Immunosuppressed Individuals with or without Progressive Multifocal Leukoencephalopathy
Source: PLoS One. 2011 Mar 16;6(3):e16736. doi: 10.1371/journal.pone.0016736 (PMC3059210; doi:10.1371/journal.pone.0016736)
Supplement: Table S5 — Samples from Immunosuppressed patients (113 samples from 90 patients). (DOC) [file pone.0016736.s005.doc]

| **Table S5**: Samples from Immunosuppressed patients (113 samples from 90 patients) | | | | | | | | | | |
| --- | --- | --- | --- | --- | --- | --- | --- | --- | --- | --- |
| Virus Name | Brain | CSF | PBMC | Plasma | Bone marrow | Whole blood | BM plasma | Blood plasma | Urine | Result source |
| KIPyV | 0/8 | 0/59 | 0/31 | 0/1 | 0/4 | 0/4 | 0/2 | 0/1 | 0/3 | Lab 1 |
|  | 0/8 | 0/59 | 0/31 | 0/1 | 0/4 | 0/4 | 0/2 | 0/1 | 0/3 | Lab 2 |
| WUPyV | 0/8 | 0/59 | 0/31 | 0/1 | 0/4 | 0/4 | 0/2 | 0/1 | 0/3 | Lab 1 |
|  | 0/8 | 0/59 | 0/31 | 0/1 | 0/4 | 0/4 | 0/2 | 0/1 | 0/3 | Lab 2 |
| MCPyV | 0/8 | 0/59 | 0/31 | 0/1 | 0/4 | 0/4 | 0/2 | 0/1 | 0/3 | Lab 1 |
|  | 0/8 | **1/59** | 0/31 | 0/1 | 0/4 | 0/4 | 0/2 | 0/1 | 0/3 | Lab 2 |

CSF: cerebral spinal fluid; PBMC: peripheral blood mononuclear cells; N/A: not available; BM: bone marrow; KIPyV: KI polyomavirus; WUPyV: WU polyomavirus; MCPyV: Merckel cell carcinoma polyomavirus.
