# Supplementary material for: Infrequent Detection of KI, WU and MC Polyomaviruses in Immunosuppressed Individuals with or without Progressive Multifocal Leukoencephalopathy
Source: PLoS One. 2011 Mar 16;6(3):e16736. doi: 10.1371/journal.pone.0016736 (PMC3059210; doi:10.1371/journal.pone.0016736)
Supplement: Table S6 — Samples from Immunocompetent patients (156 samples from 33 patients). (DOC) [file pone.0016736.s006.doc]

| **Table S6**: Samples from Immunocompetent patients (156 samples from 33 patients) | | | | | | | | | | | |
| --- | --- | --- | --- | --- | --- | --- | --- | --- | --- | --- | --- |
| Virus Name | CSF | PBMC | Plasma | Bone marrow | Whole blood | BM plasma | Blood plasma | Cell free blood plasma | BM PBMC | Urine | Result source |
| KIPyV | 0/5 | 0/35 | 0/38 | 0/9 | 0/9 | 0/6 | 0/6 | 0/1 | 0/1 | 0/44 | Lab 1 |
|  | 0/5 | N/A | N/A | 0/9 | 0/9 | 0/6 | 0/6 | 0/1 | 0/1 | 0/6 | Lab 2 |
| WUPyV | 0/5 | 0/35 | 0/38 | 0/9 | 0/9 | 0/6 | 0/6 | 0/1 | 0/1 | 0/44 | Lab 1 |
|  | 0/5 | N/A | N/A | 0/9 | 0/9 | 0/6 | 0/6 | 0/1 | 0/1 | 0/6 | Lab 2 |
| MCPyV | 0/5 | 0/35 | 0/38 | 0/9 | 0/9 | 0/6 | 0/6 | 0/1 | 0/1 | 0/44 | Lab 1 |
|  | 0/5 | N/A | N/A | 0/9 | 0/9 | 0/6 | 0/6 | 0/1 | 0/1 | 0/6 | Lab 2 |

CSF: cerebral spinal fluid; PBMC: peripheral blood mononuclear cells; N/A: not available; BM: bone marrow; KIPyV: KI polyomavirus; WUPyV: WU polyomavirus; MCPyV: Merckel cell carcinoma polyomavirus.
